# Itraconazole inhibits the Hedgehog signaling pathway thereby inducing autophagy-mediated apoptosis of colon cancer cells

**DOI:** 10.1038/s41419-020-02742-0

**Published:** 2020-07-17

**Authors:** Huiming Deng, Ling Huang, Zhongkai Liao, Mi Liu, Qiang Li, Ronghua Xu

**Affiliations:** 1 Department of Gastrointestinal Surgery, Huazhong University of Science and Technology Union Shenzhen Hospital, No. 89 Taoyuan Road, Shenzhen, 518000 China; 2 Department of General Surgery, Zengcheng District People’s Hospital of Guangzhou, No. 1 Guangming East Road, Guangzhou, 511300 China; 3 School of Hainan Provincial Drug Safety Evaluation Research Center, Hainan Medical University, No. 3 Xueyuan Road, Haikou, 571199 China; 4 Key Laboratory of Emergency and Trauma of Ministry of Education, Hainan Medical University, No. 3 Xueyuan Road, Haikou, 571199 China; 5 Department of Gastrointestinal Oncology, The First Affiliated Hospital of Hainan Medical University, No. 31 Longhua Road, Haikou, 570102 China

**Keywords:** Drug development, Drug development

## Abstract

Itraconazole is as an antifungal medication used to treat systemic fungal infections. Recently, it has been reported to be effective in suppressing tumor growth by inhibiting the Hedgehog signaling pathway and angiogenesis. In the present study, we investigated whether itraconazole induces autophagy-mediated cell death of colon cancer cells through the Hedgehog signaling pathway. Cell apoptosis and cell cycle distribution of the colon cancer cell lines SW-480 and HCT-116 were detected by flow cytometry and terminal TUNEL assay. Autophagy and signal proteins were detected by western blotting and cell proliferation-associated antigen Ki-67 was measured using immunohistochemistry. The images of autophagy flux and formation of autophagosomes were observed by laser scanning confocal and/or transmission electron microscopy. Colon cancer cell xenograft mouse models were also established. Itraconazole treatment inhibited cell proliferation via G1 cell cycle arrest as well as autophagy-mediated apoptosis of SW-480 and HCT-116 colon cancer cells. In addition, the Hedgehog pathway was found to be involved in activation of itraconazole-mediated autophagy. After using the Hedgehog agonist recombinant human Sonic Hedgehog (rhshh), itraconazole could counteract the activation of rhshh. Moreover, treatment with itraconazole produced significant cancer inhibition in HCT-116-bearing mice. Thus, itraconazole may be a potential and effective therapy for the treatment of colon cancer.

## Introduction

Colorectal cancer is a common tumor of the gastrointestinal tract ranking fourth and fifth in developed and developing countries respectively^1^. It is the second most common cancer in women (9.2%) and the third most common in men (10%)^2^. At present, colon cancer is mainly treated by surgical intervention combined with radio and chemotherapies, according to the guidelines of the National Comprehensive Cancer Network (NCCN)^3^. However, various complications of surgical resection and adverse effects of radiotherapy and chemotherapy, as well as resistance to drugs, have affected the efficacy and adherence to treatment^4^. Itraconazole is an antifungal drug of the triazole class, with a high bioavailability, broad spectrum and few side effects. It is widely used for the prevention and treatment of systemic fungal infections^5,6^. Recent studies have shown that itraconazole can induce autophagy thereby inhibiting glioblastoma growth via downregulation of steroid carrier protein 2 expression and redistribution of intracellular cholesterol^7^. Because the efflux ability of the P-glycoprotein transporter enhances the sensitivity of chemotherapy^8,9^, a clinical retrospective study showed that in patients with ovarian cancer who were treated with platinum and taxane therapy combined with itraconazole, progression-free survival and overall survival times for patients were 103 and 642 days vs 53 days and 139 days for platinum and taxane therapy alone^10^. This result suggested that itraconazole increased the chemosensitivity of cells to platinum and taxane.

Autophagy is triggered by endoplasmic reticulum stress (ERS) and unfolded protein response (UPR), when misfolded proteins accumulate and consists of degradation of proteins, cytoplasmic components and organelles within the cell which are sequestered to form bilayer or multilayered autophagosomes and then associated and degraded within lysosomes^11,12^. Though autophagy has a prosurvival function following ERS^13^, it can also induce irreversible autophagy-dependent apoptosis and autophagic cell death^14^. In adult tissue, abnormal activation of the Hedgehog signaling pathway is associated with the development of several cancer types including breast, gastric, pancreatic and ovarian cancers as well as hepatocellular carcinoma^15–18^. Binding of Sonic Hedgehog (shh) to the Hedgehog receptor protein patched homolog 1 (PTCH1) initiates activation of the Hedgehog pathway via reduction of smoothened (SMO) repression, which in turn leads to the zinc-finger transcription factor Gli family enabled transcription of downstream target genes^19^. It is known that GANT61, a small molecule inhibitor of Gli1 and Gli2, induces autophagy of human hepatocellular carcinomas^20^. Another Hedgehog pathway inhibitor vismodegib, which is an antagonist of SMO, induces autophagy of chronic myeloid leukemia cells, thereby promoting apoptosis and decreased drug resistance^21^. Studies have shown that itraconazole is not only effective for the treatment of fungal infections, but also can inhibit cancer cell growth by inactivating the Hedgehog pathway^22^. In the present study, the role of itraconazole on Hedgehog pathway related autophagy has been evaluated.

## Materials and methods

### Colon cancer cell culture techniques

Colonic cancer cell lines SW-480 and HCT-116 were sourced from the Shanghai Cell Bank of the Chinese Academy of Sciences. HT-29 colonic cancer cells were generously provided by Prof. Ling Huang (Hainan Drug Safety Evaluation Center, Hainan Medical University, Haikou, China). Cell lines were authenticated by Short Tandem Repeat (STR) profiling and examined under electron microscopy to confirm the absence of mycoplasma contamination. Cell lines were grown in Roswell Park Memorial Institute 1640 (RPMI 1640), containing supplements of fetal bovine serum (10%), penicillin (100 U/mL) and streptomycin (100 µg/mL) (Gibco Life Technologies, NY, US) in a humidified sterile incubator at 37 °C, with gaseous CO_2_ (5%) added to the atmosphere.

### Itraconazole applications

For in vitro experiments preliminary tests were performed in order to find appropriate itraconazole (Sigma-Aldrich, Lyon, France) concentrations for each measurement. The in vivo application has been adopted from a previous study^7^.

### Assay to determine cell viability

SW-480, HCT-116, and HT-29 cells were cultured in 96-well plates (5 × 10^3^ cells/well) within their logarithmic growth phase for 48 h with 0, 2.5, 5, 10, 20, 40, 60, 80, and 100 μM itraconazole. Subsequently, 10 µL of a 5 mg/mL solution of MTT (C0009, Beyotime, Biotechnology, Shanghai, China) was added to each well and left to react for 4 h. Then the medium was substituted with DMSO (150 µL/well) to dissolve the formazan dye and permit reading at an absorbance of 570 nm with an assay reader (SpectraMaxM4, MD Company, US). The IC_50_ and cell viability values (cell viability = optical density (OD) value of itraconazole-treated group/OD value of control group) were determined and SW-480 and HCT-116 cells were used for the next assay based on the measured IC_50_ values.

### Observation of cell morphology

The colonic cancer cell lines SW-480, HCT-116, and HT-29 were added to 96-well culture plates and after they reached 50–60% convergence 5 μM itraconazole and PBS were added. After incubation for 48 h, the morphology of the cells was analyzed with a microscope.

### Assay for colony formation

Initially 2000 cells were seeded in a 60 mm dish in the presence of 0.0, 2.5, 5.0, and 10.0 μM itraconazole for 48 h. Then, itraconazole was removed and cell cultivation continued for 14 days in the presence of the various indicated concentrations of itraconazole or no drug. The cells were visualized with 0.1% leucocrystal violet (219215, Sigma-Aldrich, CA, US) and the cell numbers were determined by counting random grids under a microscope. Only clusters containing >50 cells were deemed to be clones.

### Analysis of cells by flow cytometry

#### Degree of apoptosis

After washing harvested cells twice with PBS, cells were resuspended in binding buffer (500 µL) containing 5 µL of an Annexin V-FITC (Annexin V-FITC Detection Kit C1063, Beyotime Biotechnology, Shanghai, China). Then, 10 µL of propidium iodide (PI) was applied to the suspension and cells incubated for a further 10 min period. The degree of apoptosis was determined using a FACSCalibur cell analyzer (Accuri C6, Becton-Dickinson, US).

#### Analysis of cell cycle

Harvested cells were fixed in 70% ethanol at −20 °C overnight, and then washed with PBS, resuspended in staining buffer (0.5 mL) containing PI (25 µL) and RNase A (10 µL) and incubated at 37 °C for 30 min in the dark. DNA levels were subsequently measured using a FACSCalibur flow cytometer (*vide supra*).

### Western blotting

Cells and tumor tissues were lysed in RIPA buffer (P0013B, Beyotime Biotechnology, Shanghai, China). A bicinchoninic acid assay (BCA) kit (P0010S, Beyotime Biotechnology, Shanghai, China) was used to quantify the protein in a sample. Protein bands were separated by SDS-PAGE (8–14%) and then repositioned on PVDF membranes (sourced from Millipore, MA, US). After appropriate blockade, PVDF membranes were incubated with the following primary antibodies: shh (1:1000), PTCH1 (1:1000), SMO (1:1000), Gli1 (1:1000) (Cell Signaling Technology, MA, US), LC3-II (1:1000), SQSTM1 (1:1000), Beclin1 (1:2000), Atg-5 (1:5.000), Bax (1:1000), Bcl-2 (1:1000), PARP (1:1000), CDK4 (1:1000), CDK6 (1:1000), cyclin D1 (1:1000), β-Actin (1:1000) (ab51520, Abcam, MA, US); Goat anti-Rabbit IgG (1:5000) (70-GAR007, MultiSciences, Shanghai, China) and then incubated at room temperature with secondary antibodies for 60 min. Enhanced chemiluminescence chemicals (P0018, Beyotime) were employed to examine the target proteins. Recombinant human Sonic Hedgehog (rhshh) (Z03067) was purchased from GenScript, Nanjing, China. The lysosomal inhibitor chloroquine (CQ) and the autophagy inhibitor 3-methyladenine (3-MA) were purchased from Selleck Chemicals (Texas, US).

### Transmission electron microscopy observation

The SW-480 and HCT-116 cells were treated for 48 h, harvested, fixed in 2.5% pentane aldehyde, washed twice with PBS and then dehydrated in alcohol gradients. Sections were stained with uranyl acetate-lead citrate and observed using an electron microscope (Philips Co. Ltd., Netherlands); all images were captured at a magnification of 2 or 10 K.

### Fluorescence microscopy observations

SW-480 and HCT-116 cells were transfected for 24 h with adenovirus containing mRFP-GFP-tagged LC3 (tfLC3) gene, which is a marker that tracks autophagy flux (Hanbio Biotechnology Co. Ltd, Shanghai, China), washed with PBS and then exposed to itraconazole for 48 h. Treated cells were fixed in paraformaldehyde (4%) for 20 min and then washed with PBS three times. The GFP/mRFP images were acquired using an Olympus laser scanning confocal microscope (Olympus Co., Ltd., Japan).

### Xenograft tumor mice model

Procedures involving animals were performed in accordance with the Guidelines for the Humane Treatment of Laboratory Animals (Ministry of Science and Technology of the People’s Republic of China, Policy No. 2006398). All experiments were approved by the Institutional Animal Care and Treatment Committee of Hainan Medical University. Twenty-six BALB/c nude mice (females 6–8 weeks old, weight 14–16 g, purchased from Shanghai Alac Laboratory Animal Co. Ltd (License number: SCXK (Shanghai) 2017-0005) were subcutaneously injected with SW-480 and HCT-116 cells (5 × 10^6^ cells per mouse) in randomization, which was single-blinded. After tumors grew to 100–200 mm^3^ in volume in the nude mice, itraconazole liquid (75 mg/kg, b.i.d, *n* = 13) or normal saline (vehicle control, *n* = 13) was orally administered^7^ for 14 days. Then the mice were sacrificed by cervical dislocation after ether anesthesia. Subsequently, tumor weight and total weight of the nude mice were measured, and the specimens were immediately fixed in formalin or frozen at −80 °C for subsequent analysis.

### Immunohistochemical analysis

Wax embedded sliced tumor sections were de-waxed and rehydrated and inactivating endogenous peroxidase was added. Antigen was retrieved by pre-treating slides in a microwave oven with citrate buffer antigen retrieval solution (0.01 M, pH 6.0) (P0086, Beyotime Biotechnology, Shanghai, China) for 15 min. Specimens were incubated with the primary antibody at 4 °C overnight. They were then left for 30 min at ambient room temperature, washed with PBS three times and then incubated with appropriate secondary antibodies (PowerVision™ Two-Step Histostaining Reagent, PV6001, Beijing Zhong Shan-Golden Bridge Biological Technology Co., Ltd) for 15 min at 37 °C. The samples were stained with diaminobenzidine (DAB) (P0203, Beyotime Biotechnology, Shanghai, China) and counterstained with hematoxylin (H9627, Sigma, US) after which the progress of the reaction was observed with the aid of a microscope (Olympus IX73, Japan).

A minimum of five separate microscopic fields was examined to compute an average score for the intensity of immunostaining. When Ki-67 (ab92742, Abcam, MA, US) immunoreactivity was detected in the cell cytoplasm, these cells were considered to be positively stained. The degree of immunostaining (A) was classified into four categories, namely: no staining; weak staining; moderate staining; and strong staining. The score for each category was calculated thus: no staining a score of 0; weak staining a score of 1; moderate staining a score of 2; and strong staining a score of 3. The proportion of cells that were positively stained (B) was classified into 5 categories according to the percentage of intensity staining, thus: ≤5%; 6–25%; 26–50%; 51–75%; and >75%. The score for each category was: <5%, 0; 6–25%, 1; 26–50%, 2; 51–75%, 3; and >75%, 4. The total score for each examined slide was the summed scores of A+B^23,24^.

### TUNEL assay

Sections were deparaffinized and treated with proteinase K working solution at 37 °C for 20 min. Each specimen was then exposed to a mixture 5 µL TdT+45 µL fluorescein-labeled dUTP, incubated for 1 h at 37 °C and then washed with PBS three times. Next, 50 µL of converter-POD was added to the mixture for 30 min at 37 °C and then sections were again washed with PBS three times. The reaction was visualized with DAB stain (P0203, Beyotime Biotechnology, Shanghai, China) and counterstained with hematoxylin and the progress of the reaction observed with the aid of a microscope (Olympus IX73, Japan).

### Statistical methodology

SPSS Statistics for Windows (Version 21.0. Armonk, NY: IBM Corp.) was used to carry out statistical analyses. All data are represented as means ± standard deviations. The data satisfied the normality and comparisons between groups were assessed using one-way ANOVA with post hoc Bonferroni correction or Student’s *t*-test, with *P* < 0.05 considered to be a statistically significant difference.

## Results

### Itraconazole inhibited colonic cancer cell growths in vitro and in vivo

**Fig. 1:**
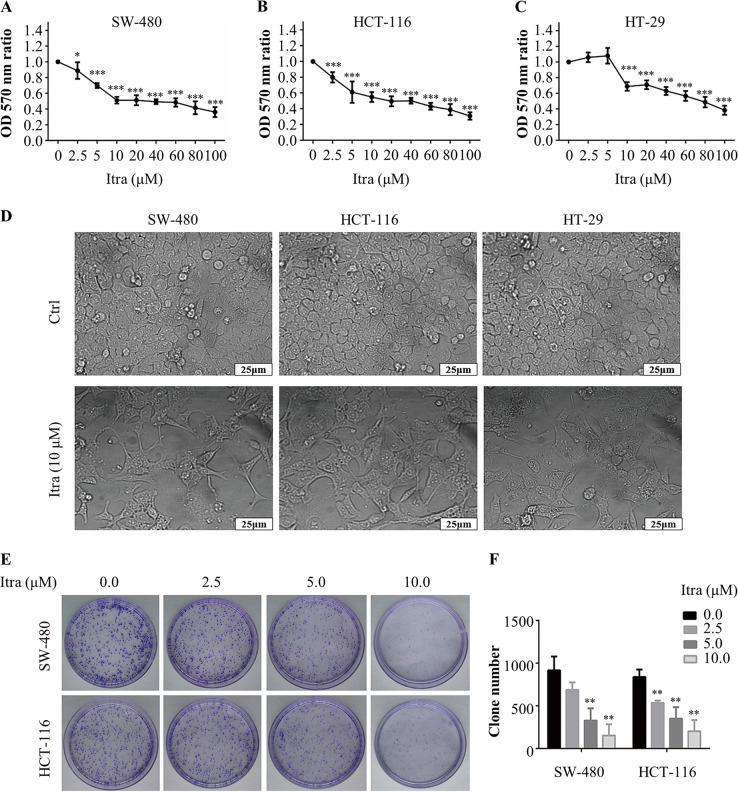
Itraconazole inhibits colon cancer cells growth in vitro. **a**–**c** SW-480, HCT-116 or HT-29 cells were treated with the indicated concentrations of itraconazole for 48 h. Cell viability measurements were performed using the MTT assay and the OD_570 nm_ ratios of itraconazole-treated/untreated cells are indicated. The data are representative of five independent experiments. **d** SW-480, HCT-116 or HT-29 cells were treated with itraconazole (5 μM) for 48 h and the cell status was observed under an inverted microscope (scale bar = 25 μm). **e**–**f** SW-480 or HCT-116 cells were consecutively treated with the indicated concentrations of itraconazole for 48 h. Then cultivation of cells was continued for 14 days and cell proliferation was examined using the colony formation assay. The data are representative of three independent experiments. *P < 0.05; **P < 0.01; ***P < 0.001 compared to the control group. Ctrl, control; Itra, itraconazole; OD, optical density.

First, we studied the potential anticancer activity of itraconazole by measuring cell growth of SW-480, HCT-116 and HT-29 human colon cancer cell lines. Each cell line was exposed for 48 h to different concentrations of itraconazole, and then MTT assays were carried out. Figure 1a–c shows that the viability of cells was decreased in a concentration-dependent manner for all cell lines examined, and IC_50_ values of SW-480, HCT-116 and HT-29 colon cancer cells were 32.42, 23.65, and 66.98 µM, respectively. In addition, we observed that the morphology of cells in the control group was normal, with smooth cell walls, good refraction, compact growth, and small intercellular spaces. In the itraconazole group, the cell structure was destroyed, the morphology was irregular, numbers were significantly reduced, cell junctions disappeared while pyknosis and other apoptotic signs appeared (Fig. 1d).

**Fig. 2:**
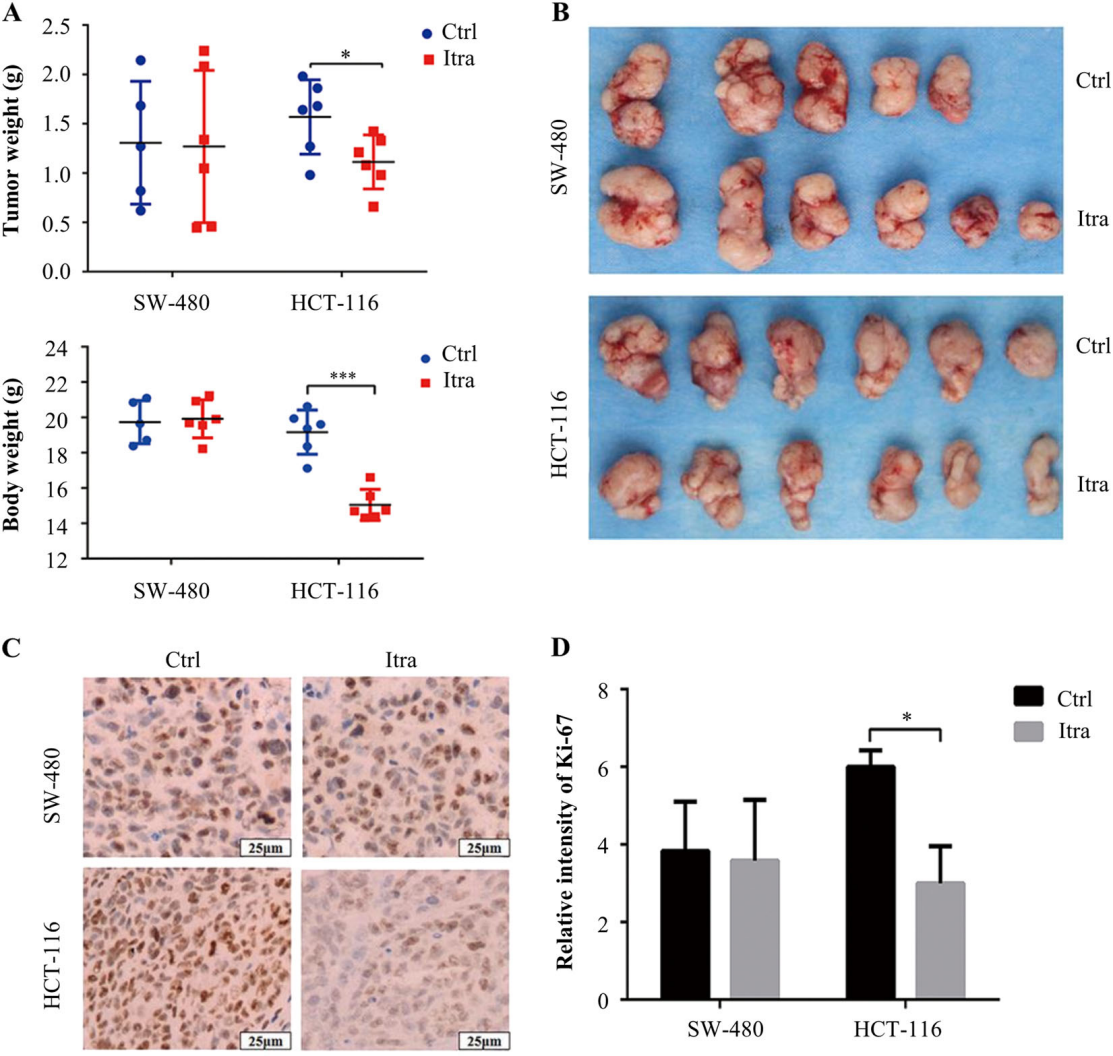
Itraconazole inhibits colon cancer cells growth in vivo. **a**, **b** Nude mice with SW-480 (Itra, *n* = 6; Ctrl, *n* = 5) or HCT-116 (*n* = 6) subcutaneous tumor xenografts were treated with normal saline (control group) or 75 mg/kg itraconazole (*n* = 6) bid by oral gavage for 14 days. During drug treatment, tumor volumes, tumor weight and total body weight were monitored every 2 day, at the end of the experiment, tumor weight and total weight of the nude mice were measured. **c**, **d** Ki-67 expression in tumors from normal saline or itraconazole-treated mice was examined by immunohistochemistry. Representative images of Ki-67 immunohistochemistry are shown (scale bar = 25 μm). *P < 0.05; ***P < 0.001 compared to the control group. Ctrl, control; Itra, itraconazole.

The colony formation assay demonstrated that itraconazole had a significant inhibitory effect on colonic cancer cells and colony numbers in cells exposed to itraconazole were markedly lower (Fig. 1e, f). The in vivo anticancer effects of itraconazole were investigated using a mouse xenograft model (two mice in the saline group and one mouse in the itraconazole group died during the 14 days of tumor growth). Itraconazole had no obvious inhibitory effect on colon cancer SW-480 cells, but it reduced significantly the rate of HCT-116 tumor growth (Fig. 2a, b). Furthermore, the proliferation marker Ki-67^25^ was expressed at much lower levels in the itraconazole-treated group compared to the controls for HCT-116 cell xenografts (Fig. 2c, d). These findings demonstrated that itraconazole markedly suppressed colonic cancer cell proliferation in vitro and for HCT-116 cells in vivo.

**Fig. 3:**
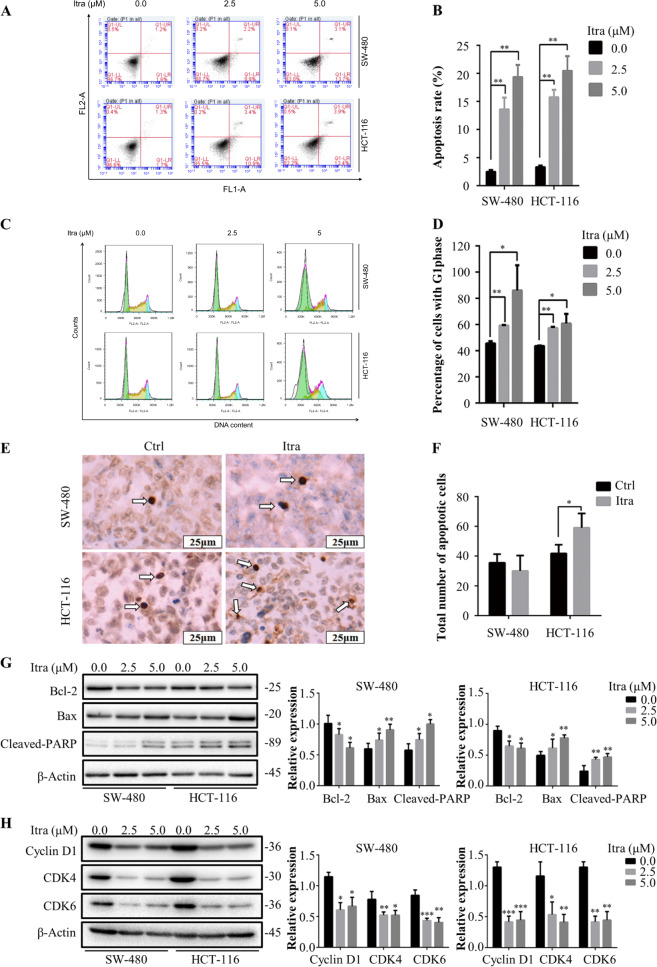
Itraconazole induces apoptosis and cell cycle G1 arrest in colon cancer cell lines. **a**–**d** SW-480 and HCT-116 cells were treated with the indicated concentrations of itraconazole for 48 h and cell apoptosis and cycle distributions were examined by flow cytometry. The data are representative of three independent experiments. **e**, **f** Apoptosis in xenograft tumor tissue examined by TUNEL assay (scale bar = 25 μm). Five different views were randomly picked for slice and the number of apoptotic cells per sample calculated as the sum of the five fields of view (*n* = 5). **g**–**h** SW-480 and HCT-116 cells were treated with the indicated concentrations of itraconazole for 48 h and apoptosis-related and cell cycle-related protein expression detected by western blotting. Protein ratios were calculated following ImageJ densitometry analysis. The data are representative of three independent experiments. *P < 0.05; **P < 0.01; ***P < 0.001 compared to the control group. Ctrl, control; FL1-A, FITC fluorescence channel; FL2-A, PI fluorescence channel; Itra, itraconazole.

To investigate whether itraconazole suppressed cell proliferation by activating apoptosis mechanisms in colonic cancer cells, we utilized flow cytometry. Figure 3a, b shows that exposure of cells to itraconazole for 48 h markedly increased the numbers of apoptotic cells compared with the controls. Similarly, we found that apoptosis in the itraconazole-treated group was significantly increased in tumor tissues of HCT-116 cells (Fig. 3e, f). In addition, we investigated the actions of itraconazole on the colon cancer cell cycle in vitro. As shown in Fig. 3c, d, itraconazole blocked the cell cycle in the G1 phase. Consistent with this observation, western blot analyses of SW-480 and HCT-116 cells revealed that expression of proteins related to apoptosis that are cleaved, e.g., PARP and Bax, were higher after itraconazole incubation than in the controls, but interestingly Bcl-2 levels were decreased in both cell lines. The levels of the cell cycle-related proteins CDK4, CDK6 and cyclin D1 were all lower after itraconazole treatment for 48 h in both cell lines (Fig. 3g, h).

### Itraconazole-induced autophagy of colonic cancer cells

**Fig. 4:**
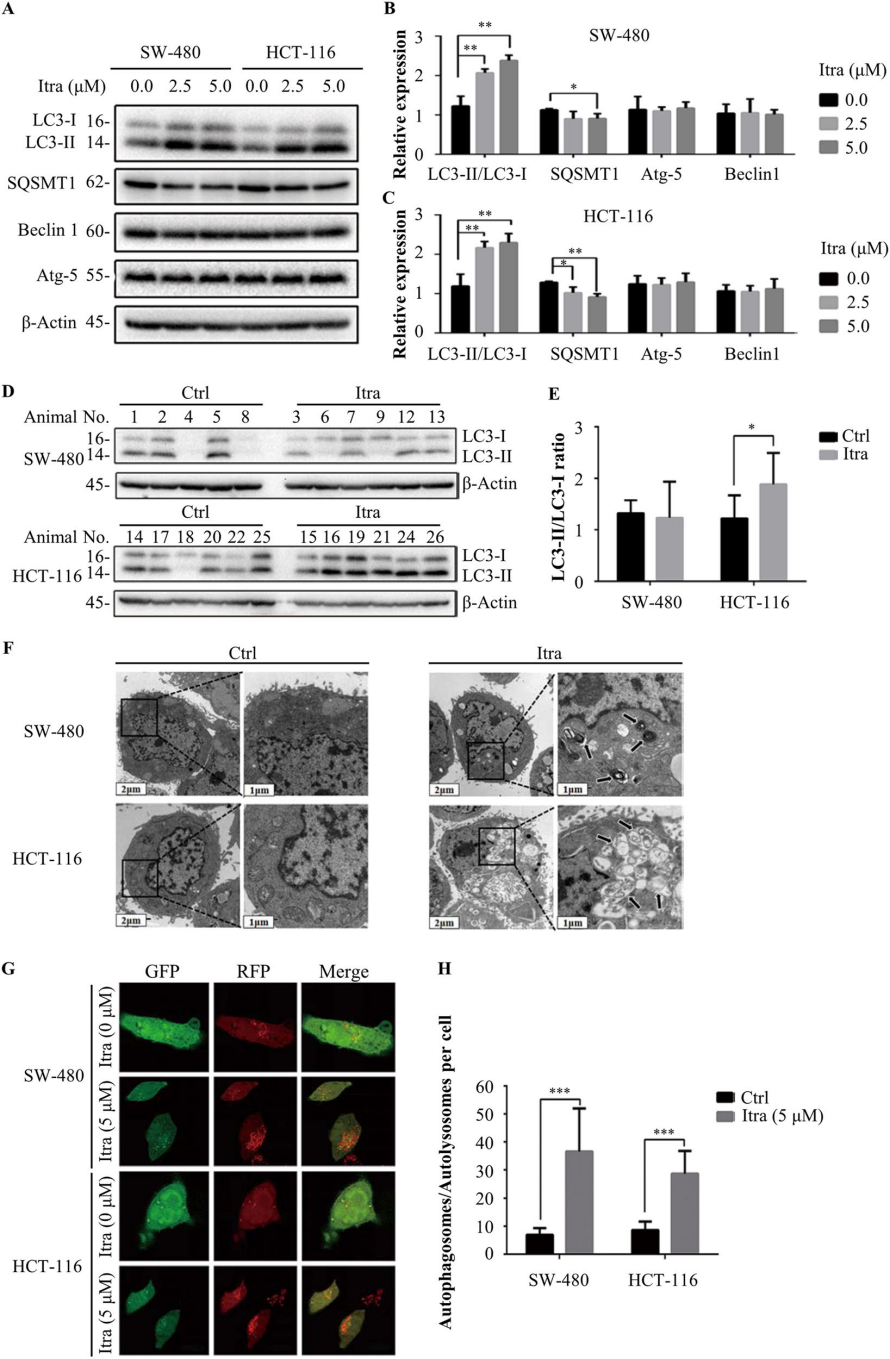
Itraconazole induces autophagy in colon cancer cells. **a**–**c** Immunoblot analysis of LC3, SQSTM1, Beclin1 and Atg-5 in SW-480 and HCT-116 cells treated with the indicated concentrations of itraconazole for 48 h. Protein ratios were calculated following ImageJ densitometry analysis. The data are representative of three independent experiments. **d**, **e** Immunoblot analysis of LC3 in SW-480 (Itra, *n* = 6; Ctrl, *n* = 5) and HCT-116 (*n* = 6) xenograft tumor tissue cells. Protein ratios were calculated following ImageJ densitometry analysis. **f** SW-480 and HCT-116 cells were treated with 0 µM or 5 µM itraconazole for 48 h, and formation of autophagosomes was examined by transmission electron microscopy analysis (scale bar = 1 μm or 2 μm). The data are representative of 3 independent experiments. **g**, **h** SW-480 and HCT-116 cells were treated with 0 or 5 µM itraconazole for 48 h and cell autophagosomes and autolysosomes were observed using laser confocal microscopy (*n* = 10). *P < 0.05; **P < 0.01; ***P < 0.001 compared to the control group. Ctrl, control; Itra, itraconazole.

Since autophagy is an important target of anticancer therapy^26^, we examined whether autophagy is induced in colonic cancer cells exposed to itraconazole. First, we detected the conversion of LC3-I to lipidated LC3-II, a marker that indicates the formation of autophagosomes^25^. Our experimental results demonstrated that itraconazole increased autophagy, as revealed by the increased conversion of LC3-II in both SW-480 and HT-116 cells. We also measured the expression of the two autophagy upregulating proteins Beclin1 and Atg-5 in SW-480 and HCT-116 colon cancer cells, but itraconazole had no detectable effects on their expression. SQSTM1 (p62) is an autophagic substrate transporter that is broken down by the autophagic pathway^27^ and its decrease was confirmed after itraconazole dose-dependent treatments of SW-480 and HCT-116 cells (Fig. 4a–c). An increased level of LC3-II in the itraconazole-treated mouse HCT-116 cell xenograft model was noteworthy, but the effect was not obvious in SW-480 cells (Fig. 4d, e). To test further for autophagy triggered by itraconazole, we looked for double-membrane autophagosomes using electron microscopy (Fig. 4f). There was a significant accumulation of both autolysosomes and autophagosomes in itraconazole treated but not control cells. However, as autophagy is a dynamic process, the increased LC3-II accumulation could suggest either autophagy enhancement or an inhibition of lysosomal degradation (that is inhibition of autophagy)^28,29^, but since also SQSTM1 (p62) was dose-dependently reduced by itraconazole (as shown in Fig. 4a–c) most likely autophagy was triggered without inhibition of lysosomal degradation.

To further demonstrate that Itraconazole promotes autophagy in colon cancer cells, we used an mRFP-GFP-LC3 adenovirus vector as a marker. As shown in Fig. 4g, h, the results indicated that itraconazole increased the yellow and red puncta, which represent autophagosomes and autolysosomes, respectively.

### Itraconazole induces apoptosis of colon cancer cells by promoting autophagy

**Fig. 5:**
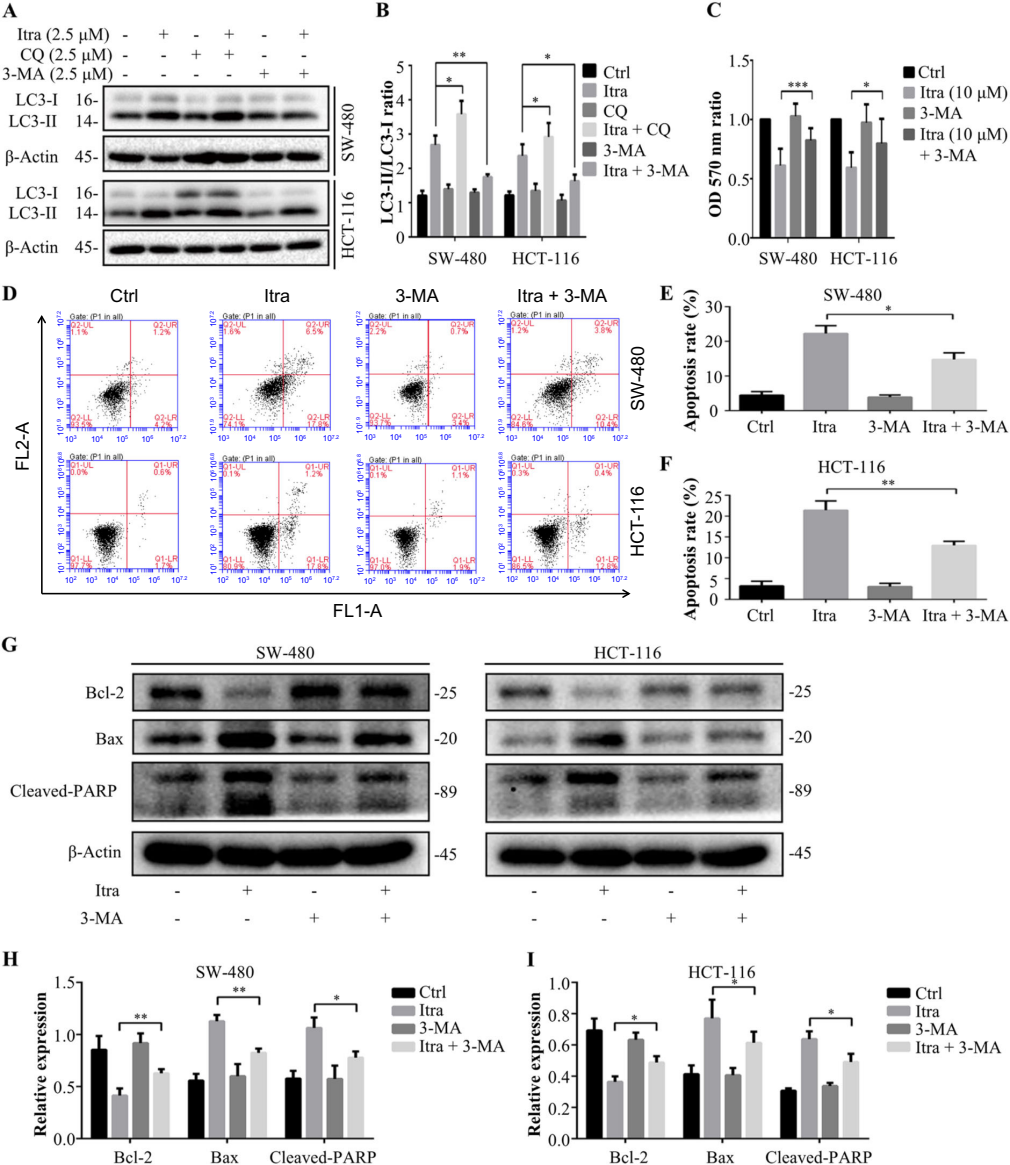
Itraconazole promoted autophagy to induce apoptosis of colon cancer cells. **a**, **b** Immunoblot analysis of LC3 in SW-480 and HCT-116 cells treated with the indicated concentrations of itraconazole for 48 h in the absence or presence of CQ or 3-MA. Protein ratios were calculated following ImageJ densitometry analysis. The data are representative of three independent experiments. **c** SW-480 and HCT-116 cells were treated with 5 µM itraconazole, 3-MA or CQ for 48 h and cell viability measured by the MTT assay. Cell viability measurements were performed using the MTT assay and the OD_570 nm_ ratios of itraconazole-treated/untreated cells are indicated. The data are representative of 5 independent experiments. **d**–**f** SW-480 and HCT-116 colon cancer cells treated with 10 µM itraconazole for 48 h in the absence or presence of 3-MA, the apoptosis rate of colon cancer cells was measured by flow cytometry. **g** SW-480 and HCT-116 colon cancer cells treated with 10 µM itraconazole for 48 h in the absence or presence of 3-MA, the expression of apoptosis-related Bcl-2, Bax and Cleaved-PARP protein were measured by immunoblot analysis and **h**, **i** protein expression ratios were calculated following ImageJ densitometry analysis. The data are representative of three independent experiments. *P < 0.05; **P < 0.01; ***P < 0.001 compared to the control group. CQ, chloroquine; Ctrl, control; FL1-A, FITC fluorescence channel; FL2-A, PI fluorescence channel; Itra, itraconazole; OD, optical density; 3-MA, 3-methyladenine.

Concomitant application of the lysosomal inhibitor chloroquine resulted in the accumulation and conversion of LC3-II. Combined treatment with the autophagy inhibitor, 3-MA markedly decreased the LC3-II conversion in itraconazole-treated colon cancer cells (Fig. 5a, b). In addition, the result of colon cancer cells treated by 3-MA combined with itraconazole showed that compared with solely itraconazole-treated cells, the viability of the colon cancer cells increased and the apoptosis rate decreased after 3-MA inhibited autophagy (Fig. 5c–f). Also the expression of apoptotic Bcl-2 protein increased and the expression of Bax and Cleaved-PARP decreased with 3-MA exposure (Fig. 5g–i). Taken together, our findings suggest that Itraconazole-induced apoptosis of colon cancer cells by promoting autophagy.

### Itraconazole inhibits the Hedgehog signaling pathway

**Fig. 6:**
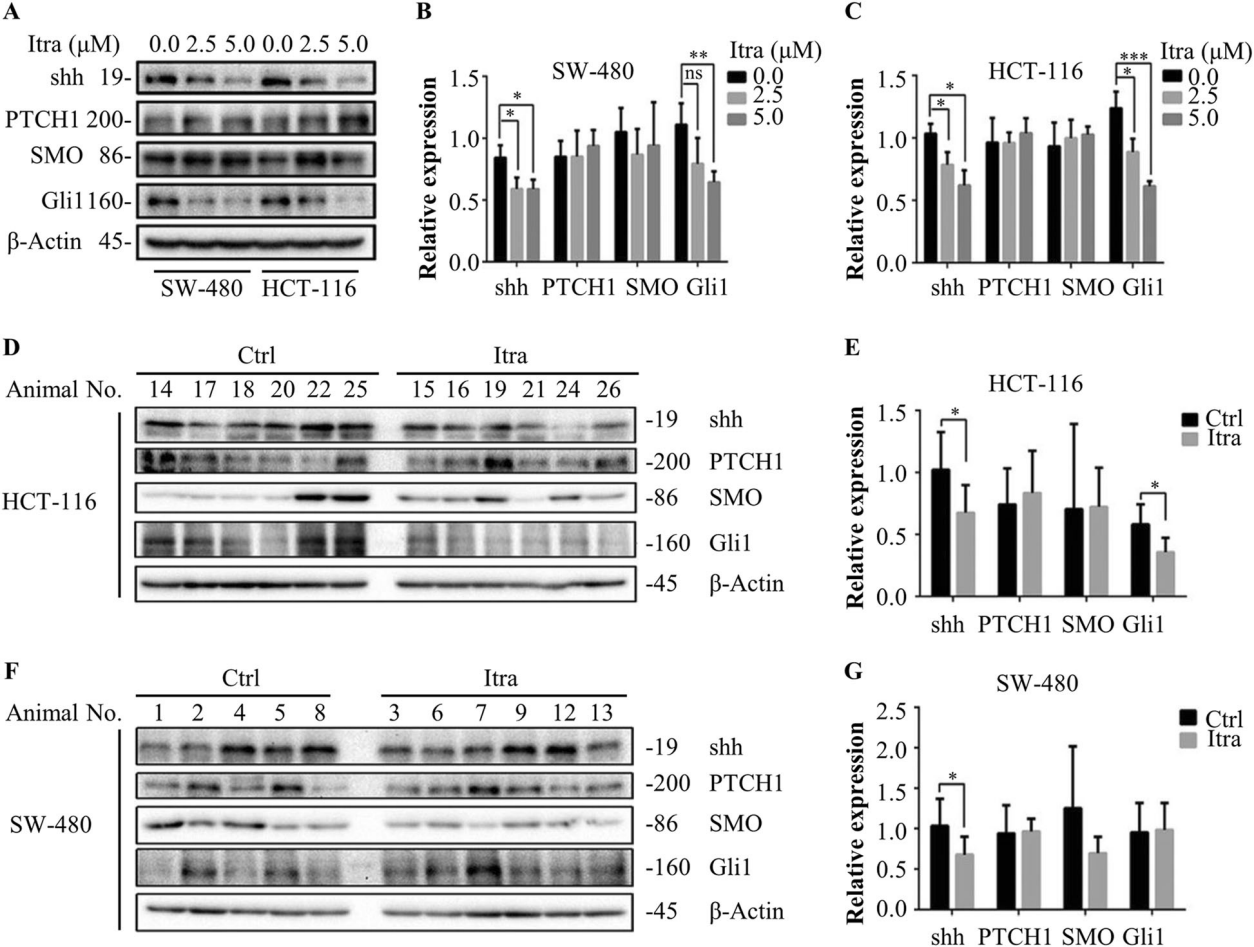
Itraconazole inhibits the Hedgehog signaling pathway. **a**–**c** Immunoblot analysis of shh, PTCH1, SMO, Gli1 in SW-480 and HCT-116 cells treated with the indicated concentrations of itraconazole for 48 h. Protein ratios were calculated following ImageJ densitometry analysis. The data are representative of three independent experiments. **d**, **e** Immunoblot analysis of shh, PTCH1, SMO, Gli1 in HCT-116 xenograft tumor cells (*n* = 6) and in (**f**, **g**) SW-480 xenograft tumor cells (Itra, *n* = 6; Ctrl, *n* = 5). Protein ratios were calculated following ImageJ densitometry analysis. *P < 0.05; **P < 0.01; ***P < 0.001 compared to the control group. Ctrl, control; Itra, itraconazole; PTCH1, patched homolog 1; SMO, smoothened.

The major components of the Hedgehog signaling pathway include shh, PTCH1, SMO and nuclear transcription factor Gli with their downstream target genes^30^. First, we examined shh, PTCH1, SMO and Gli1 levels by western blotting in SW-480 and HCT-116 cells. Figure 6a–c shows that itraconazole significantly lowered levels of shh and Gli1, but there was no difference in the expressions of PTCH1 and SMO.

There was a similar decrease in the levels of shh and Gli1 in the itraconazole-treated mouse HCT-116 cell xenograft model with also no significant difference in the expression levels of PTCH1 and SMO (Fig. 6d, e). There were decreased levels of shh and SMO in the itraconazole-treated mouse xenograft model of SW-480 cells, but there were no significant differences in the expression levels of PTCH1 and Gli1 (Fig. 6f, g).

### Itraconazole induces autophagy by decreasing Hedgehog pathway activity in colon cancer cells

**Fig. 7:**
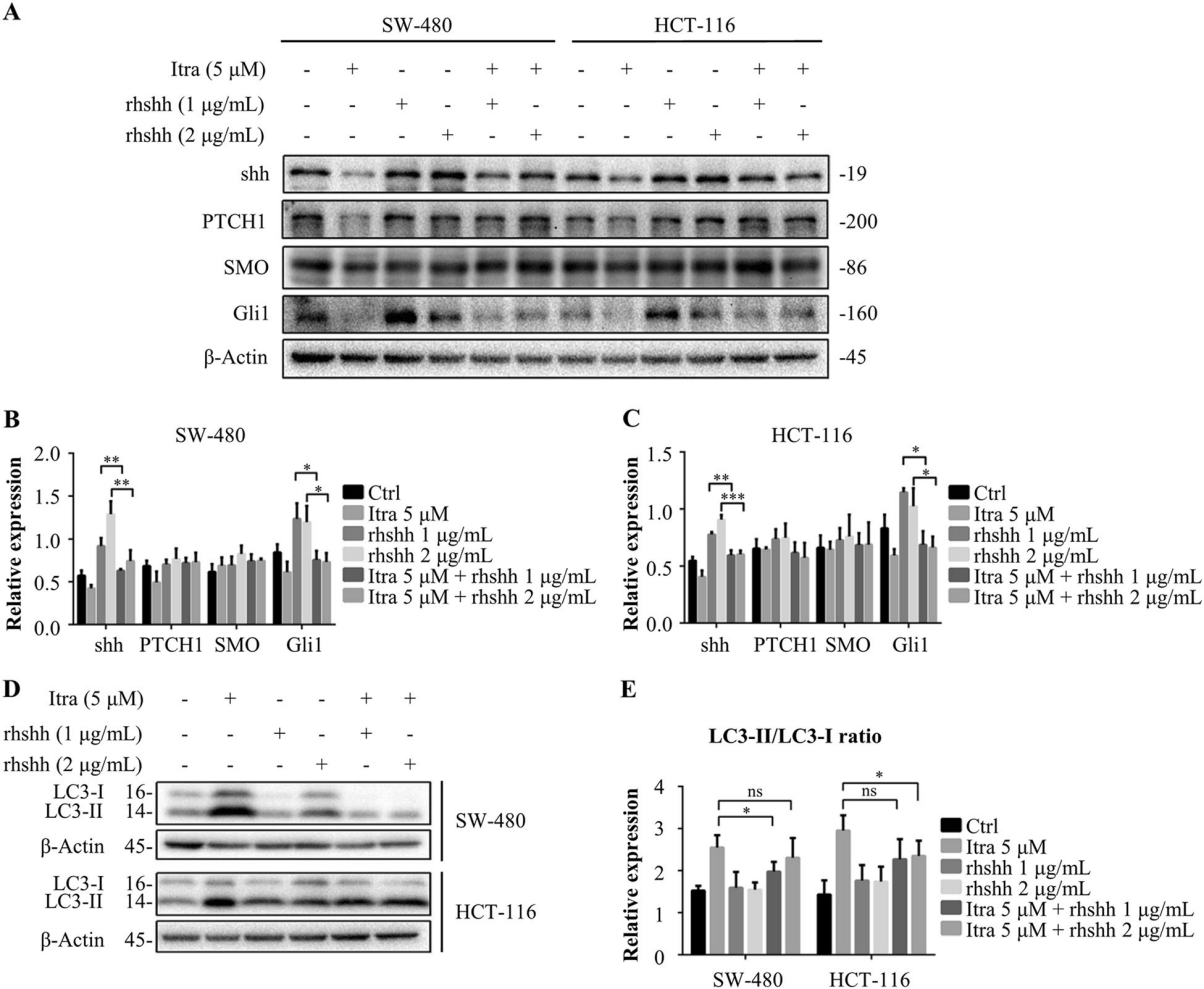
Itraconazole induces autophagy through decreasing Hedgehog pathway activity in colon cancer cells. **a**–**c** Immunoblot analysis of shh, PTCH1, SMO and Gli1 in SW-480 and HCT-116 cells treated with the indicated concentrations of itraconazole for 48 h in the absence or presence of rhshh. The data are representative of three independent experiments. **d**, **e** Immunoblot analysis of LC3 in SW-480 and HCT-116 cells treated with the indicated concentrations of itraconazole for 48 h in the absence or presence of rhshh. The data are representative of three independent experiments. Protein ratios were calculated following ImageJ densitometry analysis. The data are representative of three independent experiments. *P < 0.05; **P < 0.01 compared to the control group. Ctrl, control; Itra, itraconazole; PTCH1, patched homolog 1; rhshh, recombinant human Sonic Hedgehog; SMO, smoothened.

Next, we used rhshh at concentrations of 1 and 2 µg/mL to increase the activity of the Hedgehog pathway and examined the treatment effects of itraconazole to verify further that itraconazole could inhibit the activity of the Hedgehog pathway in SW-480 and HCT-116 cells. The results are shown in Fig. 7a–c. After rhshh stimulated the Hedgehog pathway, the expression of Gli1 and shh increased and when combined with itraconazole this effect was diminished. These findings suggested that itraconazole had an inhibitory effect on the Hedgehog pathway.

**Fig. 8:**
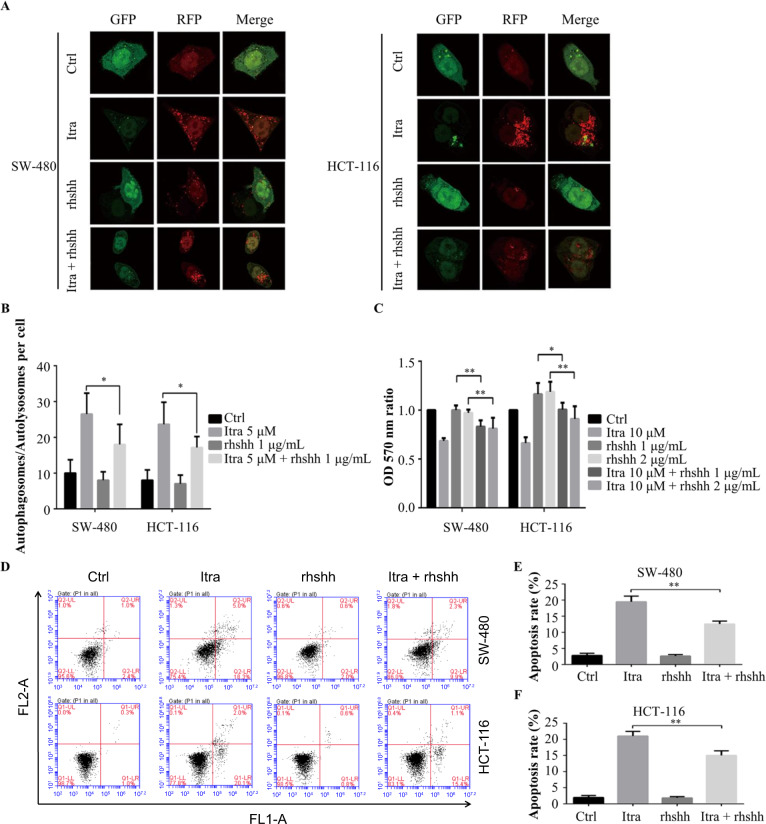
Itraconazole promotes autophagy, induces apoptosis and inhibits proliferation of colon cancer cells by inhibiting the Hedgehog pathway. **a**, **b** SW-480 and HCT-116 cells were treated with 0 or 5 µM itraconazole for 48 h in the absence or presence of rhshh and autophagosomes and autolysosomes were observed using laser confocal microscopy (*n* = 10). **c** SW-480 and HCT-116 cells were treated with 0 µM or 10 µM itraconazole for 48 h in the absence or presence of rhshh. Cell viability was examined using the MTT assay and the OD_570 nm_ ratios of itraconazole-treated/untreated cells are indicated. The data are representative of five independent experiments. **d**–**f** SW-480 and HCT-116 cells were treated with 5 µM itraconazole and for 48 h in the absence or presence of rhshh. Cell apoptosis rate was examined by flow cytometry. The data are representative of three independent experiments. *P < 0.05; **P < 0.01 compared to the control group. Ctrl, control; FL1-A, FITC fluorescence channel; FL2-A, PI fluorescence channel; Itra, itraconazole; OD, optical density; rhshh, recombinant human Sonic Hedgehog.

To determine whether Hedgehog pathway inhibition plays a role in itraconazole-induced autophagy, the Hedgehog pathway was stimulated by rhshh treatment in colonic cancer cells. Figure 7d–e shows that Hedgehog activation by rhshh resulted in much lower levels of LC3-II conversion in itraconazole-treated cells compared to the controls. In addition, we treated SW-480 and HCT-116 colon cancer cells with the same method and the results are shown in Fig. 8a, b. The number of intracellular autophagosomes after rhshh combined with itraconazole administration was significantly lower than for itraconazole treatment alone, as revealed by laser confocal scanning microscopy, suggesting that itraconazole-induced autophagy in colon cancer cells is brought about by an inhibitory effect on the Hedgehog signaling pathway. Finally, we confirmed that Hedgehog activation by rhshh resulted in markedly lower cell viability and enhanced cell apoptosis in itraconazole-treated cells compared to the controls (Fig. 8c–f).

## Discussion

The present study proved that itraconazole-induced apoptosis and G1 cell cycle arrest of SW-480 and HCT-116 cells. In vivo experiments revealed that itraconazole had inhibitory effects on HCT-116 xenografts, but SW-480 xenografts were unaffected. The possible reason might be that although itraconazole concentrations were effective in SW-480 cells cultured in vitro, due to the tumor microenvironment, immune responses and stimulating factors, doses of itraconazole used in vitro experiments, adopted from a previous study^7^, may not have been sufficient to induce inhibitory effects on SW-480 xenograft cells. This finding needs to be investigated further including prolonging the medication time and adjusting the dosage.

Prior studies showed inhibitory effect on gastric^15^, pancreatic^31^, esophageal^32^, and other cancer cells by itraconazole and that itraconazole can arrest the cell cycle heterogeneity of colon cancer cells by inhibiting Wnt and Hedgehog signaling^33^. The Hedgehog signaling pathway ligand shh interacts with PTCH1 and activated SMO and leads to Gli1 transcriptional activity, which in turn acts as a positive-feedback circuit for Hedgehog signaling^34–36^. In the absence of shh, PTCH1 inhibits SMO activity and ciliary localization^37^. Previous research proposed that the inhibitory effect of itraconazole on Hedgehog signaling is based on a direct action on SMO^22,38^. Our results demonstrated that itraconazole inhibits the expression of shh and Gli1 proteins, but did not significantly affect the expression of PTCH1 and SMO. When itraconazole was combined with the Hedgehog pathway activator rhshh, it was able to counteract the enhanced Gli1 expression induced by rhshh, and shh expression was diminished. The likely mechanism whereby itraconazole interferes with shh regulation of cell cycles might be through inhibition of the protein kinases CDK4, CDK6 and cyclin D1 expressions^39,40^. In addition, cleaved Bax and PARP levels were increased whereas Bcl2 expression decreased, which is in accordance with previously published literature, where Gli1 and Gli2 activity was shown to lead to decreased Bax/Bcl2 ratios and shh was recognized as an inducer of Bcl2 expression^41,42^. Also Ki-67 expression was reduced after itraconazole treatment, which indicated inhibition of cell-division^43^ and a previous study correlated high Ki-67 expression with Hedgehog signaling activation in pilocytic astrocytoma^44^.

However, there is a previous report which indicated, that certain anti-tumor drugs induced cell destruction by autophagy and thus enhanced anti-tumor efficacy^45^. Also, inhibition of the Hedgehog signaling pathway induced autophagy in chronic myeloid leukemia and other cancer cells^20,21,46^.

In our study, we did not detect any obvious changes in the autophagic proteins Beclin1 and Atg-5, but when we used the mRFP-GFP-LC3 adenovirus as a marker, we found that itraconazole could promote the autophagic flux of colon cancer cells, indicating that this effect might has been caused by a reduction in shh activity. However, although itraconazole reduced the expressions of the Hedgehog pathway proteins shh and Gli1, we could not exclude that other autophagy triggering factors were involved. In addition, whether autophagy was the reason for enhanced apoptosis is also not unambiguously clear, since autophagy has been reported to be a pro-survival factor in human colorectal cancer and enhances its aggressiveness as well the ability to adapt to apoptotic stimuli^47^.

## Conclusions

In conclusion, although itraconazole is an antifungal medication, our study has shown that itraconazole can effectively inhibit colon cancer cell proliferation and growth by inducing autophagy and apoptosis. Concomitantly, the expression of shh and its downstream target Gli1 protein were dose-dependently reduced by itraconazole treatment, indicating that itraconazole inhibited the Hedgehog signaling pathway. Based on these findings, itraconazole might serve as a novel adjuvant therapy for colon cancer.

## References

[1] Ferlay, J. et al. Cancer incidence and mortality worldwide: sources, methods and major patterns in GLOBOCAN 2012. *Int J. Cancer* **136**, E359–E386 (2014). 25220842 10.1002/ijc.29210

[2] Stewart, B. & Wild, C. P. *World Cancer Report 2014* (International Agency for Research on Cancer (IARC), 2014). 39432694

[3] National Comprehensive Cancer Network. NCCN clinical practice guidelines in oncology (NCCN guidelines): colon cancer (2017).

[4] Loupakis, F. et al. A review of clinical studies and practical guide for the administration of triplet chemotherapy regimens with bevacizumab in first-line metastatic colorectal cancer. *Target Oncol.* **11**, 293–308 (2016). 26687849 10.1007/s11523-015-0400-y PMC4901088

[5] Döring, M. et al. Antifungal prophylaxis with posaconazole vs. fluconazole or itraconazole in pediatric patients with neutropenia. *Eur. J. Clin. Microbiol* **34**, 1189–1200 (2015). 10.1007/s10096-015-2340-y PMC4426129 25680318

[6] Odds, F. C., Oris, M., Van Dorsselaer, P. & Van Gerven, F. Activities of an intravenous formulation of itraconazole in experimental disseminated Aspergillus, Candida, and Cryptococcus infections. *Antimicrob. Agents Ch* **44**, 3180–3183 (2000). 10.1128/aac.44.11.3180-3183.2000 PMC101627 11036047

[7] Liu, R. et al. Itraconazole suppresses the growth of glioblastoma through induction of autophagy: involvement of abnormal cholesterol trafficking. *Autophagy* **10**, 1241–1255 (2014). 24905460 10.4161/auto.28912 PMC4203550

[8] Lam, A., Hoang, J. D., Singleton, A., Han, X. & Bleier, B. S. Itraconazole and clarithromycin inhibit P-glycoprotein activity in primary human sinonasal epithelial cells. *Int Forum Allergy Rh* **5**, 477–480 (2015). 10.1002/alr.21454 25907295

[9] Pantziarka, P., Sukhatme, V., Bouche, G., Meheus, L. & Sukhatme, V. P. Repurposing drugs in oncology (ReDO)-itraconazole as an anti-cancer agent. *Ecancermedicalscience* **9**, 521–521 (2015). 25932045 10.3332/ecancer.2015.521 PMC4406527

[10] Tsubamoto, H., Sonoda, T., Yamasaki, M. & Inoue, K. Impact of combination chemotherapy with itraconazole on survival of patients with refractory ovarian cancer. *Anticancer Res.* **34**, 2481–2487 (2014). 24778064

[11] He, C. & Klionsky, D. J. Regulation mechanisms and signaling pathways of autophagy. *Annu. Rev. Gent.* **43**, 67–93 (2009). 10.1146/annurev-genet-102808-114910 PMC2831538 19653858

[12] Deegan, S., Saveljeva, S., Gorman, A. M. & Samali, A. Stress-induced self-cannibalism: on the regulation of autophagy by endoplasmic reticulum stress. *Cell Mol. Life Sci.* **70**, 2425–2441 (2013). 23052213 10.1007/s00018-012-1173-4 PMC11113399

[13] Ogata, M. et al. Autophagy is activated for cell survival after endoplasmic reticulum stress. *Mol. Cell Biol.* **26**, 9220–9231 (2006). 17030611 10.1128/MCB.01453-06 PMC1698520

[14] Levy, J. M. M., Towers, C. G. & Thorburn, A. Targeting autophagy in cancer. *Nat. Rev. Cancer* **17**, 528–542 (2017). 28751651 10.1038/nrc.2017.53 PMC5975367

[15] Hu, Q., Hou, Y.-C., Huang, J., Fang, J.-Y. & Xiong, H. Itraconazole induces apoptosis and cell cycle arrest via inhibiting Hedgehog signaling in gastric cancer cells. *J. Exp. Clin. Cancer Res.* **36**, 50–50 (2017). 28399898 10.1186/s13046-017-0526-0 PMC5387201

[16] Fattahi, S., Pilehchian Langroudi, M. & Akhavan-Niaki, H. Hedgehog signaling pathway: Epigenetic regulation and role in disease and cancer development. *J. Cell Physiol.* **233**, 5726–5735 (2018). 29380372 10.1002/jcp.26506

[17] Thayer, S. P. et al. Hedgehog is an early and late mediator of pancreatic cancer tumorigenesis. *Nature* **425**, 851–856 (2003). 14520413 10.1038/nature02009 PMC3688051

[18] Gu, D. et al. Combining hedgehog signaling inhibition with focal irradiation on reduction of pancreatic cancer metastasis. *Mol. Cancer Ther.* **12**, 1038–1048 (2013). 23468532 10.1158/1535-7163.MCT-12-1030 PMC3681871

[19] Barnfield, P. C., Zhang, X., Thanabalasingham, V., Yoshida, M. & Hui, C.-c. Negative regulation of Gli1 and Gli2 activator function by suppressor of fused through multiple mechanisms. *Differentiation* **73**, 397–405 (2005). 16316410 10.1111/j.1432-0436.2005.00042.x

[20] Wang, Y., Han, C., Lu, L., Magliato, S. & Wu, T. Hedgehog signaling pathway regulates autophagy in human hepatocellular carcinoma cells. *Hepatology* **58**, 995–1010 (2013). 23504944 10.1002/hep.26394 PMC3706478

[21] Zeng, X. et al. Targeting Hedgehog signaling pathway and autophagy overcomes drug resistance of BCR-ABL-positive chronic myeloid leukemia. *Autophagy* **11**, 355–372 (2015). 25701353 10.4161/15548627.2014.994368 PMC4502679

[22] Kim, J. et al. Itraconazole, a commonly used antifungal that inhibits Hedgehog pathway activity and cancer growth. *Cancer Cell* **17**, 388–399 (2010). 20385363 10.1016/j.ccr.2010.02.027 PMC4039177

[23] Birner, P. et al. Overexpression of hypoxia-inducible factor 1α is a marker for an unfavorable prognosis in early-stage invasive cervical cancer. *Cancer Res.* **60**, 4693–4696 (2000). 10987269

[24] Goh, V. et al. Colorectal tumor vascularity: quantitative assessment with multidetector CT—Do tumor perfusion measurements reflect angiogenesis? *Radiology* **249**, 510–517 (2008). 18812560 10.1148/radiol.2492071365

[25] Sun, Y. et al. 3′-epi-12β-hydroxyfroside, a new cardenolide, induces cytoprotective autophagy via blocking the Hsp90/Akt/mTOR axis in lung cancer cells. *Theranostics* **8**, 2044–2060 (2018). 29556372 10.7150/thno.23304 PMC5858516

[26] Fulda, S. & Kögel, D. Cell death by autophagy: emerging molecular mechanisms and implications for cancer therapy. *Oncogene* **34**, 5105–5113 (2015). 25619832 10.1038/onc.2014.458

[27] Moscat, J., Karin, M. & Diaz-Meco, M. T. p62 in Cancer: signaling adaptor beyond autophagy. *Cell* **167**, 606–609 (2016). 27768885 10.1016/j.cell.2016.09.030 PMC5114003

[28] Schaaf, M. B., Keulers, T. G., Vooijs, M. A. & Rouschop, K. M. LC3/GABARAP family proteins: autophagy-(un)related functions. *FASEB J.* **30**, 3961–3978 (2016). 27601442 10.1096/fj.201600698R

[29] Zhang, L. et al. The relevance of Nrf2 pathway and autophagy in pancreatic cancer cells upon stimulation of reactive oxygen species. *Oxid. Med. Cell Longev.* **2016**, 3897250–3897250 (2016). 26682003 10.1155/2016/3897250 PMC4670682

[30] Rimkus, T. K., Carpenter, R. L., Qasem, S., Chan, M. & Lo, H.-W. Targeting the Sonic Hedgehog signaling pathway: review of smoothened and GLI inhibitors. *Cancers* **8**, 22 (2016). 26891329 10.3390/cancers8020022 PMC4773745

[31] Jiang, F. et al. Itraconazole inhibits proliferation of pancreatic cancer cells through activation of Bak-1. *J. Cell Biochem.* **120**, 4333–4341 (2019). 30260036 10.1002/jcb.27719

[32] Chen, M. B. et al. Itraconazole-induced inhibition on human esophageal cancer cell growth requires AMPK activation. *Mol. Cancer Ther.* **17**, 1229–1239 (2018). 29592879 10.1158/1535-7163.MCT-17-1094

[33] Buczacki, S. J. A. et al. Itraconazole targets cell cycle heterogeneity in colorectal cancer. *J. Exp. Med.* **215**, 1891–1912 (2018). 29853607 10.1084/jem.20171385 PMC6028508

[34] Hui, C.-c & Angers, S. Gli proteins in development and disease. *Annu. Rev. Cell Dev. Bi.* **27**, 513–537 (2011). 10.1146/annurev-cellbio-092910-154048 21801010

[35] Scales, S. J. & de Sauvage, F. J. Mechanisms of Hedgehog pathway activation in cancer and implications for therapy. *Trends Pharm. Sci.* **30**, 303–312 (2009). 19443052 10.1016/j.tips.2009.03.007

[36] Stecca, B., Ruiz, I. & Altaba, A. Context-dependent regulation of the GLI code in cancer by HEDGEHOG and non-HEDGEHOG signals. *J. Mol. Cell Biol.* **2**, 84–95 (2010). 20083481 10.1093/jmcb/mjp052 PMC2905064

[37] Pak, E. & Segal, R. A. Hedgehog signal transduction: key players, oncogenic drivers, and cancer therapy. *Dev. Cell* **38**, 333–344 (2016). 27554855 10.1016/j.devcel.2016.07.026 PMC5017307

[38] Dirix, L. Discovery and exploitation of novel targets by approved drugs. *J. Clin. Oncol.* **32**, 720–721 (2014). 24493724 10.1200/JCO.2013.53.7118

[39] Duman-Scheel, M., Weng, L., Xin, S. & Du, W. Hedgehog regulates cell growth and proliferation by inducing Cyclin D and Cyclin E. *Nature* **417**, 299–304 (2002). 12015606 10.1038/417299a

[40] Shi, T. et al. cDNA microarray gene expression profiling of hedgehog signaling pathway inhibition in human colon cancer cells. *PLoS ONE* **5**, e13054 (2010). 20957031 10.1371/journal.pone.0013054 PMC2948497

[41] Faiao-Flores, F. et al. Targeting the hedgehog transcription factors GLI1 and GLI2 restores sensitivity to vemurafenib-resistant human melanoma cells. *Oncogene* **36**, 1849–1861 (2017). 27748762 10.1038/onc.2016.348 PMC5378933

[42] Bigelow, R. L. et al. Transcriptional regulation of bcl-2 mediated by the Sonic Hedgehog signaling pathway through gli-1. *J. Biol. Chem.* **279**, 1197–1205 (2004). 14555646 10.1074/jbc.M310589200

[43] Scholzen, T. & Gerdes, J. The Ki-67 protein: from the known and the unknown. *J. Cell Physiol.* **182**, 311–322 (2000). 10653597 10.1002/(SICI)1097-4652(200003)182:3<311::AID-JCP1>3.0.CO;2-9

[44] Rush, S. Z., Abel, T. W., Valadez, J. G., Pearson, M. & Cooper, M. K. Activation of the Hedgehog pathway in pilocytic astrocytomas. *Neuro Oncol.* **12**, 790–798 (2010). 20223881 10.1093/neuonc/noq026 PMC2940682

[45] Amaravadi, R., Kimmelman, A. C. & White, E. Recent insights into the function of autophagy in cancer. *Gene Dev.* **30**, 1913–1930 (2016). 27664235 10.1101/gad.287524.116 PMC5066235

[46] Tang, X. et al. Inhibition of Hedgehog signaling pathway impedes cancer cell proliferation by promotion of autophagy. *Eur. J. Cell Biol.* **94**, 223–233 (2015). 25824057 10.1016/j.ejcb.2015.03.003

[47] Zheng, H. Y., Zhang, X. Y., Wang, X. F. & Sun, B. C. Autophagy enhances the aggressiveness of human colorectal cancer cells and their ability to adapt to apoptotic stimulus. *Cancer Biol. Med.* **9**, 105–110 (2012). 23691463 10.3969/j.issn.2095-3941.2012.02.004 PMC3643655

